# Association of Treatment-Resistant Depression With Patient Outcomes and Health Care Resource Utilization in a Population-Wide Study

**DOI:** 10.1001/jamapsychiatry.2022.3860

**Published:** 2022-12-14

**Authors:** Johan Lundberg, Thomas Cars, Sven-Åke Lööv, Jonas Söderling, Johan Sundström, Jari Tiihonen, Amy Leval, Anna Gannedahl, Carl Björkholm, Mikael Själin, Clara Hellner

**Affiliations:** 1 Centre for Psychiatry Research, Department of Clinical Neuroscience, Karolinska Institutet, Stockholm, Sweden; 2 Stockholm Health Care Services, Region Stockholm, Stockholm, Sweden; 3 Sence Research, Uppsala, Sweden; 4 Department of Medical Sciences, Uppsala University, Uppsala, Sweden; 5 Epidemiology Division, Department of Medicine Solna, Karolinska Institutet, Stockholm, Sweden; 6 The George Institute for Global Health, University of New South Wales, Sydney, New South Wales, Australia; 7 Department of Clinical Neuroscience, Karolinska Institutet, Stockholm, Sweden; 8 Department of Forensic Psychiatry, University of Eastern Finland, Niuvanniemi Hospital, Kuopio, Finland; 9 Neuroscience Center, University of Helsinki, Helsinki, Finland; 10 Janssen-Cilag, Solna, Sweden; 11 Department of Medical Epidemiology and Biostatistics, Karolinska Institutet, Stockholm, Sweden

## Abstract

**Importance:**

The totality of the societal and individual impact of treatment-resistant depression (TRD) is unknown, as is the potential to prognosticate TRD. The generalizability of many observational studies on TRD is limited.

**Objective:**

To estimate the burden of TRD in a large population-wide cohort in an area with universal health care by including data from both health care types (psychiatric and nonpsychiatric) and, further, to develop a prognostic model for clinical use.

**Design, Setting, and Participants:**

This cohort study, a population-based observational study, assessed data from the Stockholm MDD Cohort for episodes of major depressive disorder (MDD) between 2010 and 2017 that fulfilled predefined criteria for TRD (≥3 consecutive antidepressant treatments). Data analysis was performed from August 2020 to May 2022.

**Main Outcomes and Measures:**

Outcomes were psychiatric and nonpsychiatric comorbid conditions, antidepressant treatments, health care resource utilization, lost workdays, all-cause mortality, and intentional self-harm and, in the prognostic model, TRD.

**Results:**

A total of 158 169 unipolar MDD episodes (in 145 577 patients) were identified between January 1, 2012, and December 31, 2017 (64.7% women; median [IQR] age, 42 years [30-56]). Of these, 12 793 episodes (11%) fulfilled criteria for TRD. The median (IQR) time from the start of MDD episode to TRD was 552 days (294-932). Selective serotonin reuptake inhibitor was the most common class of antidepressant treatment in all treatment steps, and 5907 patients (46.2%) received psychotherapy at some point before initiation of the third pharmacological antidepressant treatment. Compared with matched non-TRD episodes, TRD episodes had more inpatient bed-days (mean, 3.9 days; 95% CI, 3.6-4.1, vs 1.3 days; 95% CI, 1.2-1.4) and more lost workdays (mean, 132.3 days; 95% CI, 129.5-135.1, vs 58.7 days; 95% CI, 56.8-60.6) 12 months after the index date. Anxiety, stress, sleep disorder, and substance use disorder were all more common comorbid conditions in TRD episodes. Intentional self-harm was more than 4 times more common in TRD episodes. The all-cause mortality rate for patients with MDD with TRD episodes was 10.7/1000 person-years at risk, compared with 8.7/1000 person-years at risk for patients with MDD without TRD episodes (hazard ratio, 1.23; 95% CI, 1.07-1.41). Median time from start of the first antidepressant treatment to start of the second, and from start of the second antidepressant treatment to start of the third, was 165 and 197 days, respectively. The severity of MDD, defined using the self-rating Montgomery-Åsberg Depression Rating Scale (MADRS-S) at time of MDD diagnosis, was found to be the most important prognostic factor for TRD (C index = 0.69).

**Conclusions and Relevance:**

In this cohort study, TRD was a common variant of MDD when including patients from both health care types, which is associated with a high disease burden for both patients and society. The median time between initiation of new antidepressant treatments was longer than recommended in current treatment guidelines, suggesting room for more structured and timely depression care.

## Key Points

**Question:**

What is the total individual and societal impact of treatment-resistant depression (TRD) and to what extent can TRD be prognosticated at initiation of a major depressive disorder (MDD) episode?

**Findings:**

This cohort study found that, compared with patients with MDD and no TRD episodes, patients with TRD episodes had higher prevalence of psychiatric comorbid conditions, twice the utilization of outpatient health care resources, 3 times the number of inpatient bed-days, and 23% higher all-cause mortality. A prognostic model was constructed for clinical use with some discriminative capacity.

**Meaning:**

Because of the heavy individual and societal burden of TRD, early identification of patients with MDD and high risk of TRD is important for targeting health care efforts.

## Teaser

This cohort study examines data for episodes of unipolar major depressive disorder that met criteria for treatment-resistant depression to assess its impact on individuals and society and consider potential prognostic factors.

## Introduction

Major depressive disorder (MDD) is the leading cause of disability worldwide.^1^ The standard procedure to reduce symptoms and disease duration is through antidepressant treatment: antidepressant drugs (ADs), psychotherapy, and/or electroconvulsive therapy (ECT).^2^ Spontaneous remission of depressive episodes typically occurs within months or years, but a substantial number of patients do not experience a reduction in symptoms after several months and numerous treatment attempts.

The concept of treatment-resistant depression (TRD) dates to the 1970s^3^ and is typically defined operationally as a patient with MDD whose depression does not remit after 2 or more antidepressant regimens at an adequate dose and duration in the current depressive episode.^4^ Estimates from STAR*D, a prospective study of AD efficacy in MDD, imply that more than 30% of all MDD episodes could be classified as TRD episodes.^5^ Patients with TRD have residual depressive symptoms that affect functional impairment, increase their use of health care resources, and increase all-cause mortality, including an increased risk of suicide.^6^ In addition, prognostic factors for responding to the first, second, or third treatment trial, which could aid clinicians in identifying patients in need of more intense monitoring, interventions, and support,^7,8,9^ have not yet been established.^10^

Many of the observational studies on TRD to date suffer from nonrandom sampling, either due to nonuniversal access to the health care system or the exclusion of primary care data. This is a substantial limitation because the majority of patients with MDD are managed in primary care.^11,12^ Sweden has distinctive opportunities for observational research because of the country’s civic registration system^13^ and because all residents have universal access to health care^14^ with a negligible co-payment for health care visits, hospitalizations, and drugs. The record-linkage opportunities combined with the complete population health care coverage help overcome some of the limitations in data that may exist in other countries.

The aim of the present study was to estimate the patient and societal burden of TRD in a large population-wide cohort of patients with MDD, including data from both health care types (psychiatric and nonpsychiatric), and to identify factors that may help assess the risk of subsequent TRD.

## Methods

This is a population-based observational study using data from the Stockholm MDD Cohort,^15^ comprising all patients with MDD in the region of Stockholm between 2010 and 2018. In 2018, the region had an approximate population of 2.4 million inhabitants, accounting for 24% of the Swedish population. The study was approved by the regional ethics committee, Stockholm, Sweden (No: 2018/546-31), and registered at European Network of Centres for Pharmacoepidemiology and Pharmacovigilance (EU PAS Register number: 256646).^16^ All data were analyzed in a pseudonymized format, and no informed consent was required.

### Study Participants

Using Stockholm MDD Cohort data (eTable 1 in the Supplement), we identified all recorded MDD episodes^15^ that started between January 1, 2012, and December 31, 2017, in the Stockholm region, where the patient’s age was at least 18 years at start. Episodes of MDD begin with a recorded diagnosis of MDD (*International Statistical Classification of Diseases and Related Health Problems, Tenth Revision *[*ICD-10*] code F32 or F33) and end at the last recorded event related to depression (where we observed a time interval of at least 365 days after the last recorded event) (eFigure 1 in the Supplement). Each patient could contribute with 1 or more MDD episodes. To allow for analyses on comorbid conditions, health care utilization, and drug use prior to MDD episodes, patients residing in Stockholm for 12 months or less before the episode start were excluded. Episodes of MDD with records of psychosis, bipolar disorder, manic episode, or dementia were also excluded to ensure a sample of only unipolar MDD episodes (eTable 2 in the Supplement). Among the MDD episodes, we further selected episodes fulfilling predefined TRD criteria: 3 or more treatment trials (AD [Anatomical Therapeutic Chemical code N06A antidepressants], add-on medication [aripiprazole, lithium, olanzapine, quetiapine (>100 mg), risperidone], ECT, or repetitive transcranial magnetic stimulation) (eTable 3 in the Supplement). Thus, the index date for each TRD episode was the date of initiation of the third treatment trial. To qualify as a new treatment trial, an AD or add-on medication had to be initiated within the MDD episode, more than 28 days after previous treatment initiation, and have a duration of 28 days or longer. Time-interval criteria did not apply to ECT or repetitive transcranial magnetic stimulation.

Our primary study sample included all TRD episodes and matched non-TRD episodes to allow for between-group comparisons. Each TRD episode was matched, using the exact-matching method, with up to 5 non-TRD episodes based on age (within 5 years), sex, number of previous MDD episodes, and socioeconomic status.^17^ The matched non-TRD episodes were required to be initiated by antidepressant treatment and to have a duration at least as long as the time from the start of MDD until the TRD index date for the TRD episode to which they were matched. Matched non-TRD episodes were given the same index date as their matched TRD episode (eFigure 2 in the Supplement). In a sensitivity analysis, we matched all TRD episodes to up to 5 non-TRD episodes disregarding the criteria on antidepressant treatment and episode duration.

Our secondary sample was used for development of a prediction algorithm for TRD. This sample included all MDD episodes starting between January 1, 2015, and December 31, 2017, for patients residing in Region Stockholm for at least 3 years prior to the MDD episode, to ensure a sufficient time period to collect baseline data. All data analyses were performed from August 2020 to May 2022.

### Variables

The following variables were obtained for all patients in the primary study sample: age, sex, socioeconomic status,^17^ body mass index, smoking habits, number of previous MDD episodes, health care type where the MDD episode was diagnosed (psychiatric or nonpsychiatric), health care type at time of TRD, outpatient and inpatient health care utilization, lost workdays, psychiatric and somatic comorbid conditions, and antidepressant treatment (eTables 2 and 3 in the Supplement).

### Statistical Analyses

In all analyses, we estimated robust standard errors to account for the clustering structure of data unless otherwise stated. In all time-to-event analyses, MDD episodes (TRD and non-TRD episodes) were censored at the first instance of emigration from Region Stockholm, death, any of the exclusion criteria, or end of follow-up (December 31, 2018), whichever came first.

In the primary study sample, we analyzed psychiatric comorbid conditions, health care utilization, and lost workdays around the index date (−12 to +12 months). Psychiatric comorbid conditions around the index date in TRD and non-TRD episodes were presented as cumulative proportions per month (−12 to +12 months). Patients were included the first month they were diagnosed with a psychiatric comorbid condition that was carried forward in the analyses. Health care utilization was presented as the mean number of outpatient physician visits and inpatient bed-days per month (−12 to +12 months). Lost workdays were recorded as the mean number of days per month (−12 to +12 months). Analyses on lost workdays were performed for patients aged 20 to 64 years; sick-leave episodes with a duration shorter than 14 days were not included because these are paid by the employer. The association of TRD on all-cause mortality and intentional self-harm (first record of *ICD-10* codes X60-X84 after index date) was determined via Cox proportional hazard models. Intentional self-harm reported in inpatient and specialized outpatient records was included.

Treatment trials until TRD were graphically presented in an alluvial plot, including all MDD episodes in the primary study sample that fulfilled TRD criteria. The analysis is presented on a treatment-group level (selective serotonin reuptake inhibitors [SSRIs], noradrenergic and specific serotonergic antidepressant [NaSSA], serotonin and norepinephrine reuptake inhibitors [SNRIs], norepinephrine–dopamine reuptake inhibitor [NDRI], tricyclic antidepressants [TCAs], add-on medication, agomelatine, vortioxetine, other antidepressants, and ECT or repetitive transcranial magnetic stimulation) (eTable 3 in the Supplement). Time to TRD was calculated as the time from start of the MDD episode until the third treatment trial. Furthermore, we calculated the time from MDD start until initiation of first treatment trial, from first to second, and from second to third. Duration of an MDD episode was calculated as the time from the start of the episode until the end and presented in a Kaplan-Meier plot. Analyses on episode duration were only performed for patients with 1 year or longer of follow-up to enable assignment of an episode end date.

Candidate prognostic variables for TRD in the MDD cohort of the secondary study sample were defined and used as input in a risk prediction algorithm. The outcome of interest was TRD within 1 year after the start of MDD. Variables were selected based on previous reports and availability in the data^18,19,20^ (eTable 4 in the Supplement). Missingness patterns in each variable were investigated, and Montgomery-Åsberg Depression Rating Scale (MADRS-S)^21^ included 95.2% missing information, likely explained by the following: only MADRS-S ratings recorded in a structured format within 14 days before the start of the MDD episode were included, structured assessment of clinical rating scales was not fully implemented in the region during the time period, and we were only able to collect MADRS-S data covering approximately 50% of the population in Region Stockholm. We compared patients with and without records of MADRS-S and found no evidence of violations from the assumption that individuals with missing values on MADRS-S were missing at random; ie, when conditioning on variables that explain why MADRS-S is missing, the pattern of missingness was random.^22^ Thus, missing values of MADRS-S were imputed in 100 data sets using multiple imputation with chained equations, implemented in the R package mice.^23^ Lastly, we fitted a Cox proportional hazard model including all candidate prognostic variables (eAppendix in the Supplement). All data management and analyses were carried out using R version 3.6.0.

## Results

**Figure 1.**
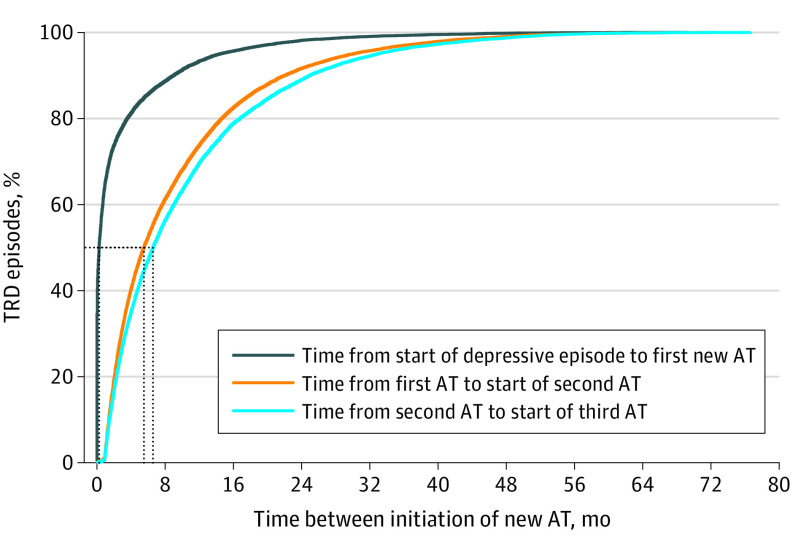
Time Between Initiation of New Antidepressant Treatment (AT) Among Patients With Episodes of Treatment-Resistant Depression (TRD)

A total of 158 169 unipolar MDD episodes (145 577 patients) were identified between January 1, 2012, and December 31, 2017 (eFigure 5 in the Supplement). Of these, 115 652 episodes (110 155 patients) had 1 or more antidepressant treatments, and 12 793 episodes (12 765 patients) fulfilled TRD criteria. The TRD episodes were matched to 62 817 non-TRD episodes, rendering a primary study sample of 75 610 episodes. Characteristics of TRD and non-TRD episodes are presented in Table 1. The median (IQR) time from the start of an MDD episode until the TRD index date was 552 days (294-932). Median time between treatment trials is presented in Figure 1. Episode duration was longer in the TRD group than in the non-TRD group (eFigure 6 in the Supplement).

**Table 1.** Characteristics at the Index Date for TRD Episodes and Matched Non-TRD Episodes

Characteristic	No. (%)	
	TRD episodes	Matched non-TRD episodes^a^
No. of episodes	12 793	62 817
No. of patients	12 765	30 380
Age at index date, y		
Median (IQR), y	43.5 (32.2-56.2)	43.4 (32.1-55.9)
18-24 y	1314 (10.3)	6292 (10.0)
≥25 y	11 479 (89.7)	56 525 (90.0)
Sex		
Women	8113 (63.4)	40 119 (63.9)
Men	4680 (36.6)	22 698 (36.1)
Depressive episode, No. (%)		
First	8204 (64.1)	40 785 (64.9)
Second	3166 (24.7)	15 472 (24.6)
Third or more	1423 (11.1)	6560 (10.4)
Time from initiation of MDD episode to TRD, d		
Mean (SD)	674 (481)	NA
Median (IQR)	552 (294-932)	NA
BMI, mean (SD)^b^	26.3 (5.6)	26.5 (5.8)
Missing	6365 (49.8)	37 407 (59.5)
Smoking habit^c^		
Yes	1355 (22.9)	4889 (20.4)
No	3574 (60.4)	14 844 (62.2)
Former	985 (16.7)	4129 (17.3)
Missing	6879 (53.8)	38 955 (62.0)
Health care type at initiation of MDD episode		
Psychiatric	4319 (33.8)	16 660 (26.5)
Nonpsychiatric	8474 (66.2)	46 157 (73.5)
Health care type at index date^a^		
Psychiatric	7740 (60.5)	17 951 (28.5)
Nonpsychiatric	5053 (39.5)	44 866 (71.5)
Psychiatric comorbid conditions^d^		
Anxiety	7564 (59.8)	27 887 (44.4)
Stress	4579 (35.8)	17 586 (28.0)
Sleep disorder	3523 (27.5)	12 178 (19.4)
Substance use disorder	1960 (15.3)	6645 (10.6)
Alcohol use disorder	1252 (9.8)	4641 (7.4)
Obsessive-compulsive disorder	511 (4.0)	2074 (3.3)
Hyperkinetic disorder	1114 (8.7)	4391 (7.0)
Autism spectrum disorder	414 (3.2)	1975 (3.1)
Personality disorder	720 (5.6)	2057 (3.3)
Intentional self-harm	624 (4.9)	1158 (1.8)
Nonpsychiatric comorbid conditions^d^		
Cardiovascular comorbidity	1087 (8.5)	5197 (8.3)
Hypertension	2472 (19.3)	11 702 (18.6)
Diabetes		
Type 1	186 (1.5)	973 (1.5)
Type 2	690 (5.4)	3501 (5.6)
Hypothyroidism	977 (7.6)	4697 (7.5)
Inflammatory bowel disease	171 (1.3)	745 (1.2)
Rheumatoid arthritis	132 (1.0)	512 (0.8)
Antidepressant therapy^e^		
Antidepressant drugs	12 786 (99.9)	62 729 (99.9)
Add-on medication	1847 (14.4)	2761 (4.4)
Psychotherapy	5907 (46.2)	21 773 (34.7)
ECT	429 (3.4)	205 (0.3)
rTMS	8 (0.1)	≤5

Abbreviations: BMI, body mass index; ECT, electroconvulsive therapy; MDD, major depressive disorder; NA, not applicable; rTMS, repetitive transcranial magnetic stimulation; TRD, treatment-resistant depression.

^a^ For TRD episodes, the index date is when the MDD episode enters into TRD. The non-TRD episodes were given the same index date as the matched TRD episode.

^b^ Calculated as weight in kilograms divided by height in meters squared.

^c^ Calculations (%) of smoking (yes/no/former) are based on episodes with nonmissing values.

^d^ Comorbid conditions recorded 5 years before the index date.

^e^ Antidepressant therapy recorded at any time before the index date.

**Figure 2.**
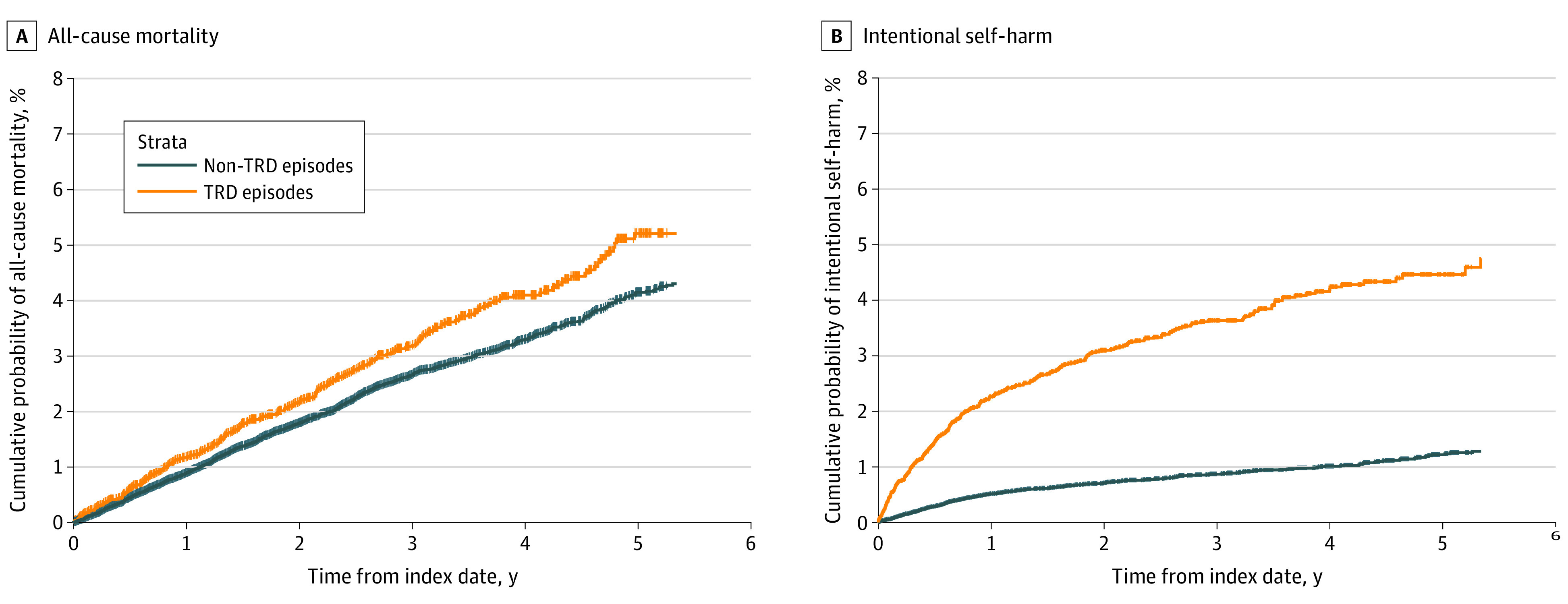
Cumulative Probability of All-Cause Mortality and Intentional Self-harm for Treatment-Resistant Depression (TRD) Episodes Compared With Matched Non-TRD Episodes The index date for TRD episodes is the date when the major depressive disorder episode fulfills TRD criteria. Non-TRD episodes were given the same index date as the matched TRD episode.

Patients with TRD episodes were more likely to have psychiatric comorbid conditions; they were more often treated with add-on medications, ECT, and psychotherapy; and their first MDD was more frequently recorded in psychiatric care, compared with patients with MDD without TRD episodes (Table 1). In patients with TRD, we observed a gradual increase in the prevalence of all psychiatric comorbid diagnoses during the year preceding the TRD index date, most pronounced for anxiety and stress (60% and 36%, respectively) (eFigure 7 in the Supplement). Table 1 also shows an increased prevalence for sleep disorders, substance abuse, personality disorders, and intentional self-harm in patients with TRD. In addition, patients with TRD episodes were more prone to increased health care resource utilization and lost workdays (eFigure 8 in the Supplement) and had a more than 50% higher mean number of outpatient physician visits 1 year before and after the index date, respectively (Table 2). For patients with TRD, the number of lost workdays was higher in the 12 months after the index date compared with before, whereas lost workdays were lower after the index date among patients with MDD without TRD episodes. The all-cause mortality rate for patients with MDD with TRD episodes was 10.7/1000 person-years at risk, compared with 8.7/1000 person-years at risk for patients with MDD without TRD episodes. Thus, TRD episodes were associated with higher all-cause mortality (HR, 1.23; 95% CI, 1.07-1.41) (Figure 2).

**Table 2.** Health Care Resource Utilization and Lost Workdays for TRD Episodes Compared With Matched Non-TRD Episodes

	Mean No. (95% CI)			
	12 mo Before index date		12 mo After index date^a^	
	TRD episodes	Matched non-TRD episodes	TRD episodes	Matched non-TRD episodes
Outpatient physician visits	11.5 (11.3-11.6)	7.3 (7.2-7.4)	9.8 (9.7-9.9)	5.6 (5.5-5.7)
Inpatient bed-days	5.7 (5.4-6.0)	1.7 (1.7-1.8)	3.9 (3.6-4.1)	1.3 (1.2-1.4)
Lost workdays	123.9 (121.3-126.5)	70.1 (68.2-72.0)	132.3 (129.5-135.1)	58.7 (56.8-60.6)

Abbreviation: TRD, treatment-resistant depression.

^a^ Including index month.

**Figure 3.**
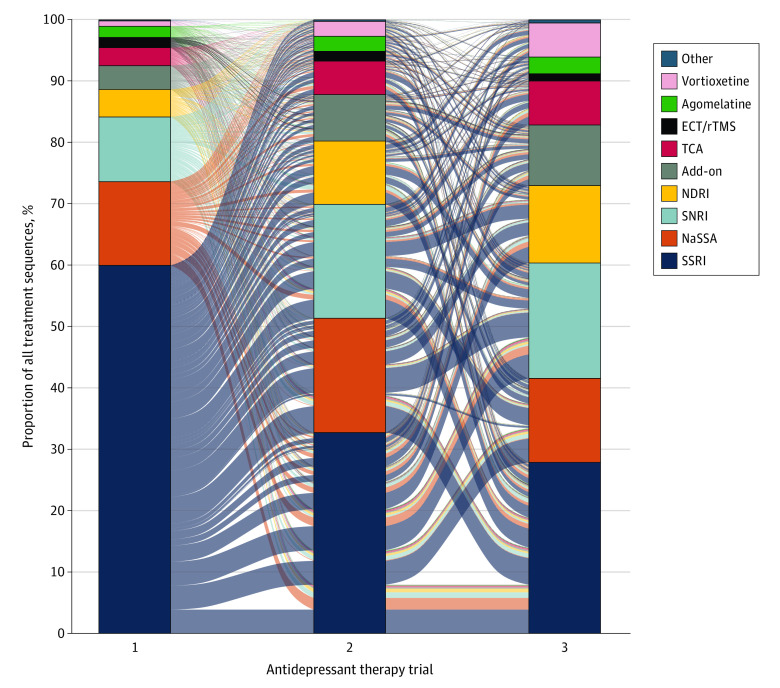
Sequence of Antidepressant Treatments Until Fulfillment of Treatment-Resistant Depression Criteria This alluvial plot presents the treatment sequence from the first to third trials of antidepressant treatment. According to the predefined criteria for treatment-resistant depression, each treatment trial is recorded within the depressive episode; that is, treatments initiated before the start of the depressive episode are not counted as a valid treatment trial because they may have been prescribed for a psychiatric condition other than depression. Codes for each group are listed in eTable 3 in the Supplement. Add-on indicates add-on medication; ECT, electroconvulsive therapy; NaSSA, noradrenergic and specific serotonergic antidepressant; NDRI, norepinephrine–dopamine reuptake inhibitor; rTMS, repetitive transcranial magnetic stimulation; SNRI, serotonin and norepinephrine reuptake inhibitor; SSRI selective serotonin reuptake inhibitor; TCA, tricyclic antidepressant.

We identified 587 different antidepressant treatment sequences until TRD criteria were reached (Figure 3). Sixty percent had SSRIs as their first treatment line, while mirtazapine (NaSSA) and bupropion (NDRI) were most common at the TRD index date (12.6% and 12.6% respectively), followed by escitalopram (SSRI) and venlafaxine (SNRI) (eFigure 9 in the Supplement). The median (IQR) time from the start of the MDD episode to initiation of the first treatment trial was 8 days (0-68), and the times from first to second and from second to third treatment were 165 days (72-375) and 197 days (84-428) (Figure 1).

A risk score for prediction of TRD was created based on all MDD episodes between January 1, 2015, and December 31, 2017 (73 056 MDD episodes and 4313 TRD episodes). Baseline characteristics for this second sample are presented in eTable 5 in the Supplement. The most important prognostic factors of TRD were MADRS-S level, health care type at MDD start, number of outpatient physician visits during the 12 months before the start of MDD, a diagnosis of sleep disorder or a filled prescription for sedatives during the past 3 years, sex, and a diagnosis of anxiety or a filled prescription for anxiolytics during the past 3 years (eFigure 11 in the Supplement). The final model is presented as a nomogram in eFigure 3 in the Supplement (a guide to using the nomogram is in eFigure 4 in the Supplement).

The final model, including 6 variables, approximated 95.0% of the full model and yielded a C index of 0.69; internal bootstrap validation indicated only minimal overfitting, and the risk score was well calibrated (eFigure 10 in the Supplement). To evaluate the prognostic value of MADRS-S, we performed a sensitivity analysis that only included patients with records of MADRS-S, and MADRS-S was still the most important prognostic factor (C index = 0.67).

## Discussion

This comprehensive population-wide study based on the Stockholm MDD Cohort showed that patients with MDD with TRD episodes had substantially higher risks of intentional self-harm, all-cause mortality, health resource utilization, lost workdays, and psychiatric comorbidities such as anxiety and stress, compared with patients with MDD without TRD episodes. In accordance with previous studies,^24^ we also found that MDD episodes with TRD were substantially longer than MDD episodes without TRD.

The proportion of patients with MDD with TRD episodes was 11.1%, which is lower than the 30% reported in the STAR*D trial^25^ and slightly lower than the 13% to 15% reported in observational studies in Sweden and Denmark.^9,26^ A likely reason that the registry-based studies have lower proportions of patients with TRD, compared with the STAR*D study, is the substantially lower switch rate in clinical practice with more than 550 days in our study to reach the third treatment, compared with approximately 134 days (19.1 weeks) in STAR*D.^25^ Another reason for the lower proportion of TRD in our study could be that we also included patients from primary care. In the present study, most MDD diagnoses were initially recorded in primary care, but TRD occurred mainly after transfer to psychiatric care, which is expected because regional guidelines recommend referral only for those with at least 2 previous treatment trials unless there are complicating factors.^27^ In contrast to previous studies,^9,25^ we did not impose a restriction for time to TRD. The median time to the third treatment trial (TRD index date) was approximately 550 days; there is a Taiwanese study in which the recorded time to TRD is twice as long.^28^ The time between initiation of the first and second treatments and between the second and third (median, 165 and 197 days, respectively) is a confirmation of previously observed antidepressant treatment durations in Sweden^29^ and Europe.^30^ This long duration is contrasted by the findings that nonresponse can be reliably predicted 2 weeks after initiating an AD and that median time to remission in clinical trials typically is 8 weeks or less.^24,27,31^ Current treatment guidelines recommend a sequenced treatment approach with early assessment and adjustment of treatment, including switching within weeks if a response is not achieved. There are several plausible scenarios that may contribute to a long time between treatment trials, including an inadequate assessment of the response. In the present study, patients with MDD with TRD episodes have higher rates of lost workdays both the year before and after the TRD index date, so it is reasonable to assume that sustained remission has not been achieved.

We show that having TRD was associated with a more than 20% increase in all-cause mortality, which is in alignment with previous studies.^9,32,33^ Although causes of death were not investigated, we found that factors closely linked to risk of suicide, such as self-harm, were higher in patients with TRD, especially during the year following the index date. This has also been observed in other studies.^9,32^ The somatic comorbidity was not increased, which makes somatic conditions a less likely explanation for the higher mortality. Psychiatric comorbidities such as fatigue, anxiety disorders, and obsessive-compulsive disorders were more common among patients with MDD in a TRD episode, compared with patients with MDD without TRD episodes, which is in line with previous findings,^34^ and substance abuse was also higher among these patients, as has been found by others.^9,18^ Treatment-resistant depression has previously been associated with increased loss of workdays and absenteeism, contributing to the large societal and individual burden associated with MDD.^35,36^ In the present study, the average monthly number of lost workdays was more than twice as high for patients with TRD episodes during the index month and up to 12 months after, indicating a sustained presence of disability. Treatment-resistant depression has been associated with high health care resource utilization.^7,8^ We found that the average number of health care visits (outpatient and inpatient) both before and after the index date was higher among patients with MDD in TRD episodes.

Many different ADs were prescribed for patients with TRD, with SSRIs being the most common class. As a first treatment, this would be expected because it adheres to Swedish guidelines. More surprisingly, SSRIs and other monoamine reuptake inhibitors were also dominant in later treatment lines, where other treatments such as augmentation with atypical antipsychotic or lithium have shown efficacy and are also recommended for use.^37^ Recently, the failure of psychotherapy has been suggested for inclusion in the concept of TRD,^38^ and although it was not counted as a formal treatment trial for TRD status, around 50% of patients in the current cohort have been given psychotherapy and could be considered treatment resistant also with regards to psychotherapy. Further studies should investigate how including psychotherapy in the definition of TRD affects outcomes. Data show that other nonpharmacological treatments, such as repetitive transcranial magnetic stimulation^39^ or ECT, were rarely prescribed for TRD, even though ECT is readily available in the region.

Several risk factors for the development of TRD have been identified, including age, psychiatric comorbidities, disease duration, age at onset, history of abuse, and treatment-related factors.^18,37^ However, in the present study, only severity of depression at diagnosis (MADRS-S) was shown to be a strong prognostic factor for TRD, which suggests the importance of severity assessment by using clinical rating scales. Many of the other prognostic factors contributed but to a minor extent. A limitation both for clinical depression care and for our prognostic model was the large proportion of patients who did not have data for MADRS-S. To maximize the generalizability of our model, we used multiple imputation as our primary approach. This strategy was corroborated by similar results seen in the sensitivity analysis comprising a selected subsample of all patients with MADRS-S data (complete case analysis).

Structured assessment of the response through use of rating scales has been shown to improve treatment outcomes substantially, by providing timely updates of progress to prompt treatment adjustments.^40^ Previous studies have shown that measurement-based care, where patients are followed up in a structured way evaluating the effect of treatment, is more efficacious.^40,41^ However, the analysis is based on retrospective data, and no randomization was used for treatment allocation. Thus, these data cannot tell us if any particular treatment may decrease the risk for developing TRD.

### Strengths and Limitations

The examination of a population with access to universal health care and inclusion of all patients with MDD from all health care types are strengths because they allow assessment of the burden of depression for a full population. The number of TRD episodes and patients with TRD were almost the same, likely explained by the limited follow-up time for identifying several TRD episodes for most patients. Our main goal for focusing on episodes rather than patient number was to maximize the number of data points in our study.

Relying on a definition of TRD based on prescription patterns and diagnoses alone introduces uncertainties. For example, the definition relies on clinicians to identify non-response and prescribe a new treatment when nonresponse is identified, and records reflect only patients adhering to the prescribed treatment. Patients who for some reason do not persist with treatment or never receive treatment in the first place are not classified as TRD. A previous study found that approximately 25% of patients who started an antidepressant medication stopped treatment without achieving remission or switching^42^; however, the size of this group cannot be determined without a structured follow-up. Given the long time it takes for patients to fulfill TRD criteria in our study, these patients reflect a subpopulation of patients who persisted in treatment. Importantly, this is also true for the matched patients with MDD without TRD. Moreover, not all patients who would qualify for a diagnosis seek treatment, and patients who start treatment may drop out without clinical improvement. Despite the above uncertainties, we show that the patients with TRD we identify have worse outcomes on virtually all parameters investigated.

All variables investigated in the prediction model had low proportions of missing values, except for MADRS-S, allowing a longitudinal analysis. The operational definition of initiation of a new AD treatment did not include the interpretation of clinical rating scales but an assumption that switches made after day 28 were due to lack of effect. This approach may have led to an overestimation of the number of true patients with TRD but is justified by the knowledge that adverse effects typically emerge in the beginning of a treatment and later switches are mainly due to persistence or re-emergence of depressive symptoms. The ability of the present study to include registered lost workdays is very important, particularly because societal costs account for approximately 85% of all costs associated with MDD.^43,44^

## Conclusions

This study provides a reliable estimate of prevalence of patients with MDD who develop TRD in Stockholm, Sweden, a region with universal access to health care, regardless of health care type (psychiatric or nonpsychiatric). The findings show that TRD was associated with higher disease burden with respect to health care resource utilization, lost workdays, intentional self-harm, and mortality and that these findings are not explained by MDD duration. The long duration from MDD diagnosis until TRD suggests that clinicians could improve their alignment to recommendations of timely follow-up of patient symptoms. Our finding that the risk of subsequent TRD can be assessed by severity could help clinicians identify at first MDD diagnosis the patients in need of closer follow-up.

## Notes

Supplement. **eTable 1.** Stockholm MDD Cohort **eTable 2.** Definitions of covariates added to the primary study sample **eTable 3.** Definitions of treatment groups **eTable 4.** Definition of predictor variables included in the development of the prediction model **eTable 5.** Baseline characteristics the secondary study sample (for development of the prediction model) **eAppendix.** Statistics: Prognostic Model **eFigure 1**. Definition of MDD episode **eFigure 2.** Matching TRD episodes to non-TRD episodes **eFigure 3.** Nomogram for the final prognostic model **eFigure 4.** Nomogram example **eFigure 5.** Flow chart for the derivations of the primary study sample **eFigure 6.** Duration of the depressive episode **eFigure 7.** Psychiatric comorbid conditions among TRD and matched non-TRD episodes **eFigure 8.** Monthly healthcare resource utilization and lost work-days for TRD episodes compared to matched non-TRD episodes **eFigure 9.** Treatments (on a substance level) when patients fulfill the TRD criteria (N=12 793) **eFigure 10.** Calibration of the prediction model **eFigure 11.** Importance of each variable in the full model measured by partial χ^2^ minus the predictor degrees of freedom
