# Supplementary material for: Association of Treatment-Resistant Depression With Patient Outcomes and Health Care Resource Utilization in a Population-Wide Study
Source: JAMA Psychiatry. 2022 Dec 14;80(2):167–75. doi: 10.1001/jamapsychiatry.2022.3860 (PMC9856735; doi:10.1001/jamapsychiatry.2022.3860)
Supplement: Supplement. — eTable 1. Stockholm MDD Cohort eTable 2. Definitions of covariates added to the primary study sample eTable 3. Definitions of treatment groups eTable 4. Definition of predictor variables included in the development of the prediction model eTable 5. Baseline characteristics the secondary study sample (for development of the prediction model) eAppendix. Statistics: Prognostic Model eFigure 1. Definition of MDD episode eFigure 2. Matching TRD episodes to non-TRD episodes eFigure 3. Nomogram for the final prognostic model eFigure 4. Nomogram example eFigure 5. Flow chart for the derivations of the primary study sample eFigure 6. Duration of the depressive episode eFigure 7. Psychiatric comorbid conditions among TRD and matched non-TRD episodes eFigure 8. Monthly healthcare resource utilization and lost work-days for TRD episodes compared to matched non-TRD episodes eFigure 9. Treatments (on a substance level) when patients fulfill the TRD criteria (N=12 793) eFigure 10. Calibration of the prediction model eFigure 11. Importance of each variable in the full model measured by partial χ2 minus the predictor degrees of freedom [file jamapsychiatry-e223860-s001.pdf]

Supplemental Online Content

Lundberg J, Cars T, Lööv SA, et al. Association of treatment-resistant depression with patient outcomes and health care resource utilization in a population-wide study. *JAMA Psychiatry*. Published online November 23, 2022.
doi:10.1001/jamapsychiatry.2022.3860

eTable 1. Stockholm MDD Cohort

eTable 2. Definitions of covariates added to the primary study sample

eTable 3. Definitions of treatment groups

eTable 4. Definition of predictor variables included in the development of the prediction model

eTable 5. Baseline characteristics the secondary study sample (for development of the prediction model)

eAppendix. Statistics: prognostic model

eFigure 1. Definition of MDD episode

eFigure 2. Matching TRD episodes to non-TRD episodes

eFigure 3. Nomogram for the final prognostic model

eFigure 4. Nomogram example

eFigure 5. Flow chart for the derivations of the primary study sample

eFigure 6. Duration of the depressive episode

eFigure 7. Psychiatric comorbid conditions among TRD and matched non-TRD episodes

eFigure 8. Monthly healthcare resource utilization and lost work-days for TRD episodes compared to matched non-TRD episodes

eFigure 9. Treatments (on a substance level) when patients fulfill the TRD criteria (N=12 793)

eFigure 10. Calibration of the prediction model

eFigure 11. Importance of each variable in the full model measured by partial χ^2 minus the predictor degrees of freedom

This supplemental material has been provided by the authors to give readers additional information about their work.

© 2022 Lundberg J et al. *JAMA Psychiatry.*

eTable 1. Stockholm MDD Cohort

The Stockholm MDD Cohort (SMC)[1] comprises all patients with MDD in the region of Stockholm between 2010 and 2018 and includes data from the following data sources:

VAL	EHR	SSIA
The Stockholm regional healthcare data warehouse (called VAL) includes information on all contacts with healthcare financed by the Region Stockholm. Data for primary care, secondary care and hospitalizations are available from the 1980s (inclusion of diagnoses from primary care from 2003). The International Classification of Diseases Version 10 (ICD-10) has been used since 1997. VAL also contains demographic information on patient age, sex, migration status and death. Information on prescription drugs dispensed in the ambulatory setting have been included since July 2010. The drug dispensing data come from the same source as the Swedish Prescribed Drug Register, with a population coverage of over 99%. The Anatomical Therapeutic Chemical (ATC) classification system is used to code dispensed drugs. [2]	Stockholm with its 2.4 million citizens accounts for 24% of the Swedish population. TakeCare is presently the most widely used EHR system in the healthcare region of Stockholm. The EHR installation in the Stockholm Healthcare Region has over 50,000 active users representing several groups of healthcare professionals. The installation handles more than 4 million patient records and covers more than 88% of inpatient care and 75% of outpatient care. [3]	The registries held at the Swedish Social Insurance Agency gives a basis for longitudinal statistics and research about entire populations or groups, and employment and alternative employments (studies, parental leave, unemployment, labour market activities, etc) as well as gainful employment and illness. [4]

1. Lundberg J, Cars T, Lööv SÅ, et al. Clinical and societal burden of incident major depressive disorder: A population-wide cohort study in Stockholm. *Acta Psychiatr Scand*. Published online February 15, 2022. doi:10.1111/acps.13414

2. Zarrinkoub R, Wettermark B, Wändell P, et al. The epidemiology of heart failure, based on data for 2.1 million inhabitants in Sweden. *Eur J Heart Fail*. 2013; 15: 995- 1002.

3. Cars T. Real-Time Monitoring of Healthcare Interventions in Routine Care: Effectiveness and Safety of Newly Introduced Medicines. http://uu.diva-portal.org/smash/get/diva2:1015130/FULLTEXT01.pdf. Accessed September 06, 2022.

4. Everhov ÅH, Khalili H, Askling J, et al. Sick leave and disability pension in prevalent patients with Crohn's disease. *J Crohns Colitis.* 2018; 12: 1418- 1428.

© 2022 Lundberg J et al. *JAMA Psychiatry.*

eTable 2. Definitions of covariates added to the primary study sample

Condition	Included codes
Psychiatric conditions	
MDD diagnosis	ICD10:<br>F32 – Depressive episode<br>F33 - Recurrent depressive disorder
Psychosis	ICD10:<br>F20-F29 Schizophrenia, schizotypal and delusional disorders
Manic episode	ICD10:<br>F30 Manic episode
Bipolar disorders	ICD10:<br>F31 Bipolar affective disorder
Dementia	ICD10:<br>F00 – Dementia in Alzheimer disease<br>F01 – Vascular dementia<br>F02 – Dementia in other diseases classified elsewhere<br>F03 – Unspecified dementia
Anxiety	ICD10:<br>F41– Other anxiety disorders
OCD	ICD10:<br>F42– Obsessive compulsive disorder
Stress	ICD10:<br>F43– Reaction to severe stress, and adjustment disorders (excl PTSD F43.1)
Substance use	ICD10:<br>F10-F19 - Mental and behavioral disorders due to psychoactive substance use
Sleep disorders	ICD10:<br>G47 – Sleep disorders<br>F51 - Nonorganic sleep disorders
Personality disorders	ICD10:<br>F60 - Specific personality disorders
Hyperkinetic disorders	ICD10:<br>F90 - Hyperkinetic disorders
ASD – Autism spectrum disorders	ICD10:<br>F840 - Childhood autism<br>F841 - Atypical autism<br>F845 - Asperger syndrome
Intentional self-harm	ATC:<br>X60-X84 – Intentional self-harm
Non-psychiatric conditions	
Cardiovascular disease	ICD10:<br>I20-I25, I50, I110, I42 (excl I42.1 and I42.2), I43, I48, I60-I64, G45, I70-I72, I73.1, I73.9, I74, I77.3, I77.6, I77.8, I79
Hypertension	ICD10:<br>I10-I15
Diabetes mellitus type II	ICD10:<br>E11 - Diabetes mellitus type II
Rheumatoid arthritis	ICD10:<br>M05 - Seropositive rheumatoid arthritis<br>M06 - Other rheumatoid arthritis
Inflammatory bowel disease	ICD10:<br>K50 – Crohn disease [regional enteritis]<br>K51 - Ulcerative colitis
Hypothyroidism	ICD10:<br>E03 - Other hypothyroidism

© 2022 Lundberg J et al. *JAMA Psychiatry.*

eTable 3. Definitions of treatment groups

Treatment group	Definition	Included codes
Antidepressants (AD)	Antidepressants	ATC:<br>N06A
SSRI	selective serotonin reuptake inhibitors	ATC:<br>N06AB
NASSA	noradrenergic and specific serotonergic antidepressant	ATC:<br>N06AX11 - mirtazapine<br>N06AX03 – mianserin
SNRI	serotonin and norepinephrine reuptake inhibitors	ATC:<br>N06AX16 - venlafaxine<br>N06AX17 - milnacipran<br>N06AX21 - duloxetine<br>N06AX23 – desvenlafaxine
NDRI	norepinephrine–dopamine reuptake inhibitor	ATC:<br>N06AX12 – bupropion
ADD-ON	Add-on medications	ATC:<br>N05AH03 - olanzapine<br>N05AH04 - quetiapine<br>N05AX08 - risperidone<br>N05AX12 - aripirazole<br>N05AN01 – lithium
TCA	tricyclic antidepressants	ATC:<br>N06AA02 – imipramine<br>N06AA04 – clomipramine<br>N06AA06 – trimipramine<br>N06AA07 – lofepramine<br>N06AA09 – amitryptiline<br>N06AA10 – nortriptyline<br>N06AA21 – maprotiline
Agomelatine	Agomelatine	ATC:<br>N06AX22 – agomelatine
Vortioxetine	Vortioxetine	ATC:<br>N06AX26 – vortioxetine
ECT or rTMS	Electroconvulsive therapy	Clinical procedure codes:<br>DA006, DA024, DA025 - ECT<br>DU050 - rTMS
Other antidepressants (other)	Other	ATC:<br>N06AX02 – tryptophan<br>N06AX05 – trazodone<br>N06AX06 – nefazodone<br>N06AX14 – tianeptine<br><br>N06AF03 – phenelzine<br>N06AF04 – tranylcypromine<br>N06AG02 – moclobemide<br>N06AX18 – reboxetine
Psychotherapy	Psychotherapy	Clinical procedure codes:<br>DU008-DU011, DU013, DU020-DU023

© 2022 Lundberg J et al. *JAMA Psychiatry.*

eTable 4. Definition of predictor variables included in the development of the prediction model

**Variable**	**Definition**
Age	Age at index i.e., age at initiation of the MDD episode
Sex	Men/Women
Number of depressive episodes	This variable indicates in which MDD episode the patients currently are in (i.e., if the variable is equal to 3 this means that the patient has had two previous depressive episodes in their disease history (data available from 1 January 1997)
Healthcare level	This variable presents the healthcare level where the first MDD diagnosis within the current MDD episode was recorded. In order to make this variable as simple as possible we divided this variable into two levels only: (1) Non-psychiatric care and (2) psychiatry care. The level non-psychiatric mainly comprise patients diagnosed in primary healthcare settings.
Treated in psychiatric care	This binary variable (yes/no) presents if the patient within three years before index (start of current MDD episode) has been treated in psychiatric healthcare.
Treatment with antidepressants	This binary variable (yes/no) presents if the patient within three years before index (start of current MDD episode) has been treated with antidepressants (ATC: N06A)
Treatment with add-on medication	This binary variable (yes/no) presents if the patient within three years before index (start of current MDD episode) has been treated with add-on medication (olanzapine, quetiapine, risperidone, aripiprazole or lithium)
Treatment with ECT/rTMS	This binary variable (yes/no) presents if the patient within three years before index (start of current MDD episode) has been treated with either ECT or rTMS.
Treatment with psychotherapy	This binary variable (yes/no) presents if the patient within three years before index (start of current MDD episode) has been treated with psychotherapy.
Psychiatric comorbid conditions	
Sleep disorders	This binary variable indicates if the patient within the past three years before initiation of current MDD episode either has been diagnosed for sleep disorders (ICD10:G47 or F51) or at least one filled prescription of a sedative (ATC: N05C)
Anxiety	This binary variable indicates if the patient within the past three years before initiation of current MDD episode either has been diagnosed for anxiety (ICD10:F41) or at least one filled prescription of an anxiolytic (ATC: N05B)
Substance use	This binary variable indicates if the patient within the past three years before initiation of current MDD episode either has been diagnosed with mental and behavioral disorders due to psychoactive substance use (ICD10:F10-F19).
Obsessive compulsive disorder - OCD	This binary variable indicates if the patient within the past three years before initiation of current MDD episode been diagnosed for OCD (ICD10:F42)
Personality disorder	This binary variable indicates if the patient within the past three years before initiation of current MDD episode been diagnosed with personality disorder (ICD10:F60)
Hyperkinetic disorders	This binary variable indicates if the patient within the past three years before initiation of current MDD episode been diagnosed for hyperkinetic disorders (ICD10:F90)
Autism spectrum disorder - ASD	This binary variable indicates if the patient within the past three years before initiation of current MDD episode been diagnosed with autism spectrum disorder (ICD10:F840, F841, F845)
Personality disorders	This binary variable indicates if the patient within the past three years before initiation of current MDD episode been diagnosed for personality disorder (ICD10:F60)
Intentional self-harm	This binary variable indicates if the patient within the past three years before initiation of current MDD episode has a recording of intentional self-harm (ICD10:X60-X84). We included diagnosis of intentional self-harm recorded both in outpatient and inpatient care.
Non-psychiatric comorbid conditions	
Cardiovascular disease	This binary variable indicates if the patient within the past three years before initiation of current MDD episode been diagnosed with cardiovascular disease (ICD10: I20-I25, I50, I110, I42 (excl I42.1 and I42.2), I43, I48, I60-I64, G45, I70-I72, I73.1, I73.9, I74, I77.3, I77.6, I77.8, I79)
Hypertension	This binary variable indicates if the patient within the past three years before initiation of current MDD episode been diagnosed with hypertension (ICD10: I10-I15)

© 2022 Lundberg J et al. *JAMA Psychiatry.*

Diabetes mellitus type I	This binary variable indicates if the patient within the past three years before initiation of current MDD episode been diagnosed with hypertension (ICD10: E10)
Diabetes mellitus type II	This binary variable indicates if the patient within the past three years before initiation of current MDD episode been diagnosed with hypertension (ICD10: E11)
Rheumatoid arthritis - RA	This binary variable indicates if the patient within the past three years before initiation of current MDD episode been diagnosed with RA (ICD10: M05, M06)
Inflammatory bowel disease - IBD	This binary variable indicates if the patient within the past three years before initiation of current MDD episode been diagnosed with IBD (ICD10: K50, K51)
Hypothyroidism	This binary variable indicates if the patient within the past three years before initiation of current MDD episode been diagnosed with hypothyroidism (ICD10: E03)
Healthcare utilization and work-loss	
Lost workdays	This continuous variable includes the number of lost workdays during the past 12 months before index (initiation of current MDD episode). We included both sick-leave days and days with disability pension.
Outpatient physician visits	This continuous variable includes the number of outpatient physician visits during the past 12 months before index (initiation of current MDD episode).
Clinical rating scales	
MADRS-S	This variable includes the most recent recorded MADRS-S score before index (initiation of current MDD episode). In the development of the prediction model, we only included MADRS-S recorded within 14 days before index. Patients with missing information on MADRS-S were imputed using multiple imputation (see methods section)

© 2022 Lundberg J et al. *JAMA Psychiatry.*

eTable 5. Baseline characteristics the secondary study sample (for development of the prediction model)

	All unipolar MDD episodes initiated between 1 January 2015 and 31 December 2017
N	73,056
**Demographics**	
Age (at initiation of MDD episode)	
- Median (IQR)	42.0 (30.0–57.0)
Sex (% women)	64.0%
**Depressive episode (%)**	
- First depressive episode	66.9%
- Second depressive episode	23.1%
- Third+ depressive episode	9.9%
**Healthcare level at start of the current MDD episode**	
- Non-psychiatric care	76.9%
- Psychiatric care	23.1%
**Treatment in psychiatric care**	
- Treated in psychiatric healthcare before start of MDD episode	31.8%
**Psychiatric comorbid conditions**	
- Diagnosis of sleep disorder or treatment with sedatives	37.7%
- Diagnosis of anxiety or treatment with anxiolytics	44.9%
- Diagnosis of stress (excluding PTSD)	23.5%
- Diagnosis of intentional self-harm	1.1%
- Diagnosis of OCD	1.5%
- Diagnosis of Autism spectrum disorders	1.5%
- Diagnosis of personality disorder	1.4%
**Non-psychiatric comorbid conditions**	
- Diagnosis of cardiovascular disease	7.9%
- Diagnosis of hypertension	16.9%
- Diagnosis of diabetes mellitus type I	1.2%
- Diagnosis of diabetes mellitus type II	5.0%
- Diagnosis of hypothyroidism	6.0%
- Diagnosis of inflammatory bowel disease	1.0%
- Diagnosis of rheumatoid arthritis	0.7%
**Healthcare utilization**	
- Mean (SD) outpatient physician visits during the past year prior to initiation of MDD episode	6.3 (6.9)
**Work-loss**	
- Mean (SD) days of work-loss during the past 12 months prior to initiation of MDD episode	35.6 (88.4)
**Antidepressant therapy prior to initiation of MDD episode**	
- Antidepressants (ATC: N06A)	39.8%
- Add-on medication	1.6%
- ECT/rTMS	0.0%
- Psychotherapy	14.0%
**Clinical rating scales**	
- MADRS-S (mean, SD)	26.3 (8.5)

This table is based on data from the first imputed dataset.
Index: Time of initiation of MDD episode

© 2022 Lundberg J et al. *JAMA Psychiatry.*

eAppendix. Statistics: prognostic model

Candidate prognostic variables for TRD in the MDD-cohort of the secondary study sample were defined and used as input in a risk prediction algorithm. The outcome of interest was TRD within one year after start of MDD. Variables were selected based on previous reports and availability in the data (eTable 4). Missingness patterns in each variable was investigated and Montgomery Åsberg Depression Rating Scale (MADRS-S) included 95.2% missing information, likely explained by the fact that: only MADRS-S recorded in a structured format within 14 days prior the start of the MDD-episode were included, structured assessment of clinical rating scales was not fully implemented in the region during the time period, and that we were only able to collect MADRS-S data covering approximately 50% of the population in Region Stockholm. We compared patients with and without records of MADRS-S and found no evidence of violations from the assumption that individuals with missing values on MADRS-S were missing at random (MAR); i.e., when conditioning on variables that explain why MADRS-S is missing, the pattern of missingness was random[21]. Thus, missing values of MADRS-S were imputed in 100 datasets using multiple imputation with chained equations, implemented in the R-package "mice". Lastly, we fitted a Cox's proportional hazard model including all candidate prognostic variables. To create a clinically useful model, we approximated the full model by using a fast backward algorithm on an ordinary least squares model in which the estimated linear predictor from the full Cox model was the outcome and all candidate variables were entered in exactly the same manner as in the full Cox model. Thus, in the first step $R^2 = 1.0$ by design, and by removing variables in a stepwise manner the full model could be approximated to an arbitrary level. The model was internally validated using 300 bootstrap samples. Discrimination was assessed by Harrell's *c*-index and calibration was assessed graphically by comparing observed event rates with the predicted risk at one and two years after index. The final model was presented as a nomogram (eFigures 3 and 4). In a sensitivity analysis, we used the same model but only including patients with MADRS-S (i.e., complete case analysis). We performed this sensitivity analysis to evaluate if the prognostic value of MADRS-S was similar in the two fitted models.

eFigure 1. Definition of MDD episode

Since we do not have clinical depression ratings scales recorded at regular timepoints for all patients, we established a definition in order to get a proxy for the length of an MDD episode (or rather, the duration of healthcare contacts related to MDD). This was operationally defined based on recorded activities related to depression:

(1) For each patient we selected the patient's first recorded MDD-diagnosis (ICD10: F32-Depressive episode and F33-Recurrent depressive episode), initiating the first documented depressive episode (i.e., the index date)
(2) For each patient we further analyzed the time from first recorded MDD-diagnosis to subsequently recorded events related to depression. If the time interval between the recording of depressive events was ≤365 days, the episode was categorized as ongoing while if the time interval was >365 days, the episode was categorized as closed at the date of the last recorded depressive event. As depressive events, we allowed (1) recording of depression diagnoses, (2) filled prescriptions of antidepressants (AD; ATC: N06A) and add-on medication for depression (lithium, risperidone, olanzapine, aripiprazole, and quetiapine (>100 mg)), electroconvulsive therapy (ECT), repetitive transcranial magnetic stimulation (rTMS) or treatment with psychotherapy. If the last recorded depressive event was a dispensation of either AD or add-on medication, we extended the episode with the number of dispensed tablets (i.e., a maximum of 100 days was added)
(3) When an episode is categorized as closed, a subsequent recording of a depression diagnosis code would initiate the start of a new episode).

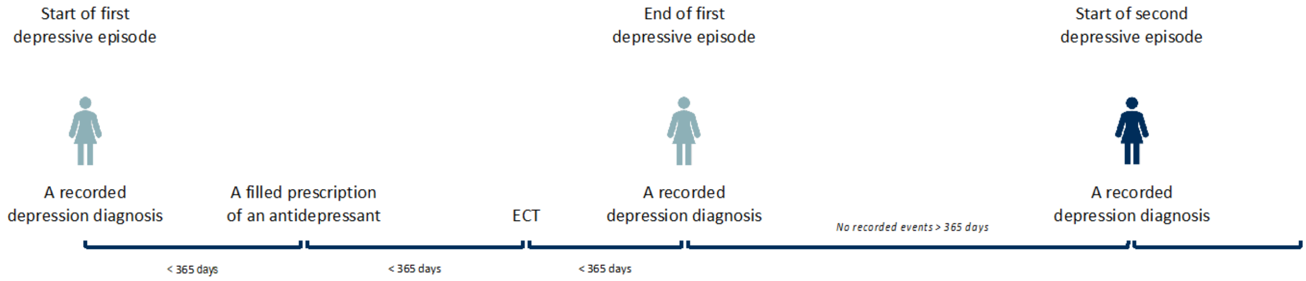

© 2022 Lundberg J et al. *JAMA Psychiatry.*

eFigure 2. Matching TRD episodes to non-TRD episodes

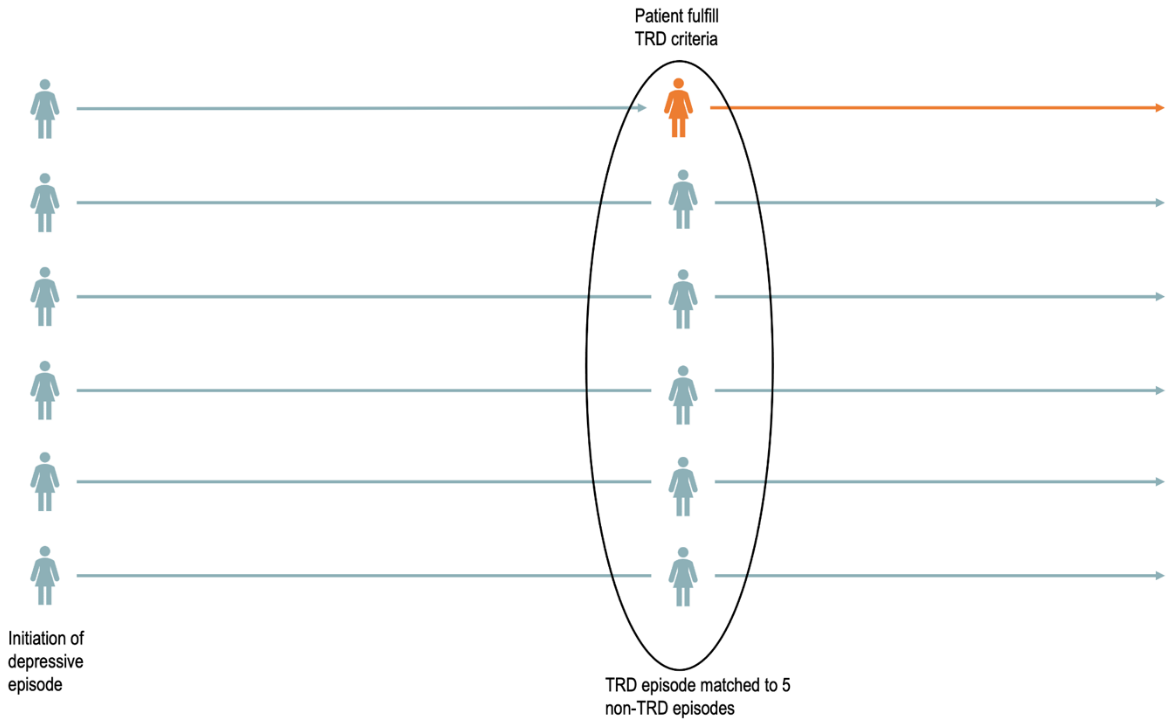

Each MDD episode that fulfill TRD criteria will be matched to a non-TRD depression episode on age, sex, socioeconomic status, and length of depressive episode. This means that a TRD episode can only be matched to a non-TRD episode having a duration that corresponds to the time when the matched TRD episode fulfills TRD criteria (i.e., the non-TRD episode must be ongoing at the time when the matched TRD-episode fulfill the TRD criteria). The matched non-TRD episode will be given the same index as the matched TRD episode. This means that if a patient enters into TRD 200 days after initiation of the depressive episode, the TRD index will be start of depressive episode + 200 days. The five matched non-TRD episodes will be given the same index i.e., start of depressive episode + 200 days.

© 2022 Lundberg J et al. *JAMA Psychiatry.*

eFigure 3. Nomogram for the final prognostic model

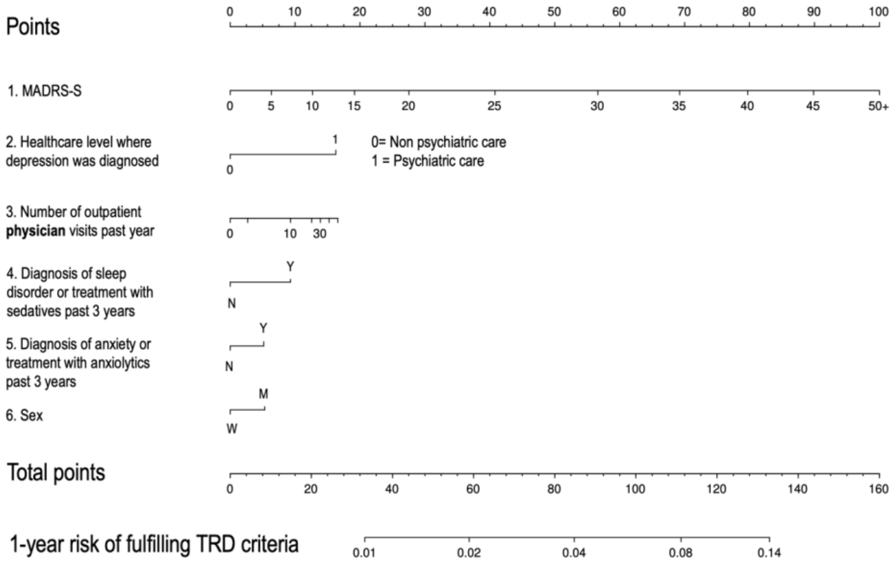

© 2022 Lundberg J et al. *JAMA Psychiatry.*

eFigure 4. Nomogram example

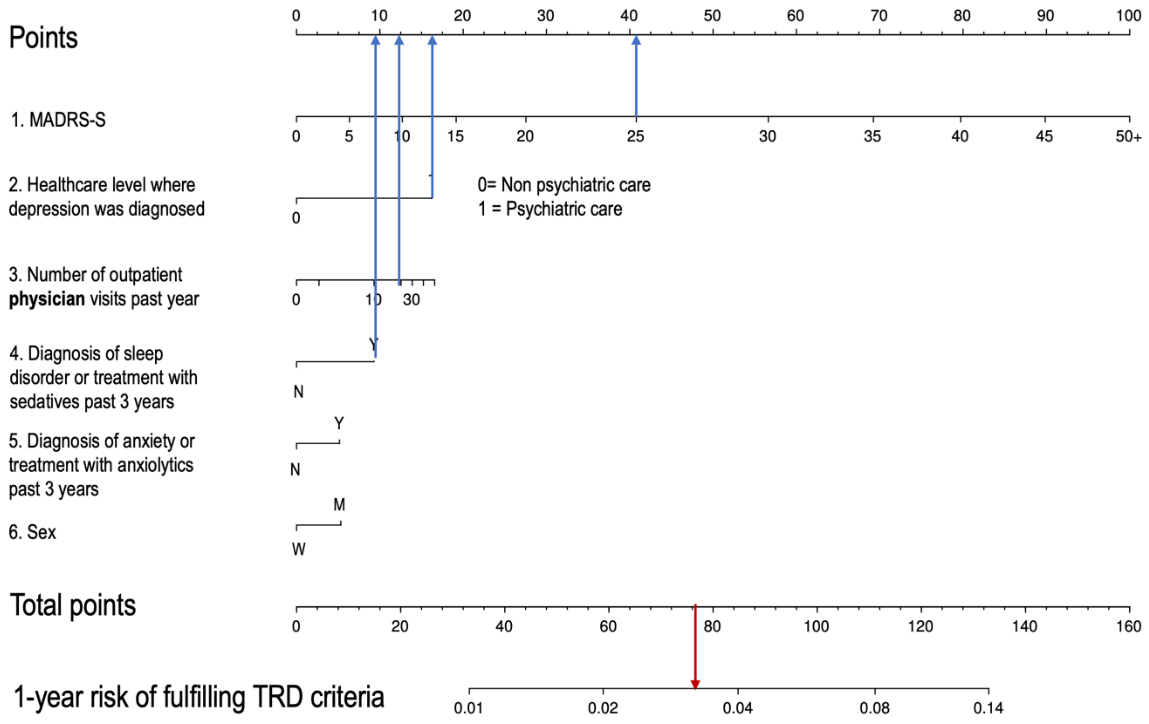

A woman with an MDD episode initiated in psychiatric care (16 points), a MADRS-S score of 25 (41 points), previous use of sedatives (8 points) and twenty outpatient physician visits during the past 12 months (12 points) will have a total score of 16+41+8+12=77 points and a 1-year risk of fulfilling TRD criteria of approximately 3.3%.

© 2022 Lundberg J et al. *JAMA Psychiatry.*

eFigure 5. Flow chart for the derivations of the primary study sample

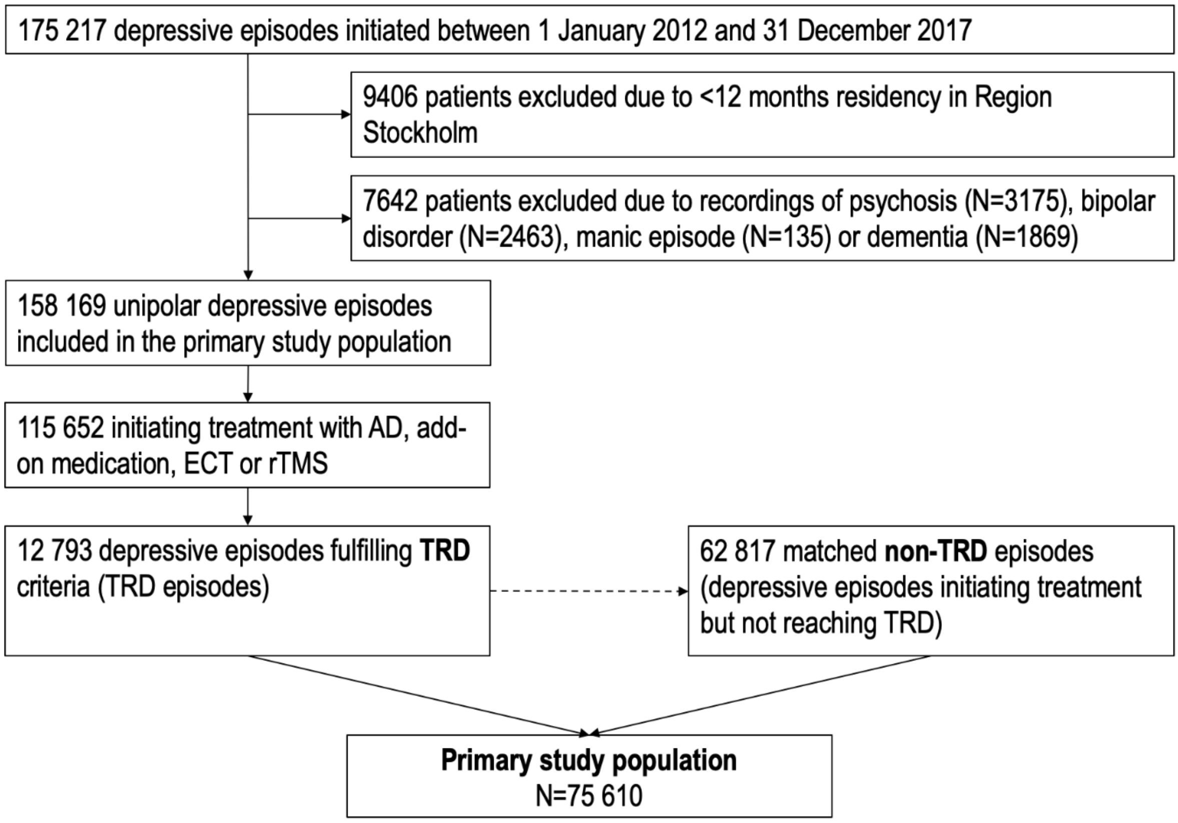

© 2022 Lundberg J et al. *JAMA Psychiatry.*

eFigure 6. Duration of the depressive episode

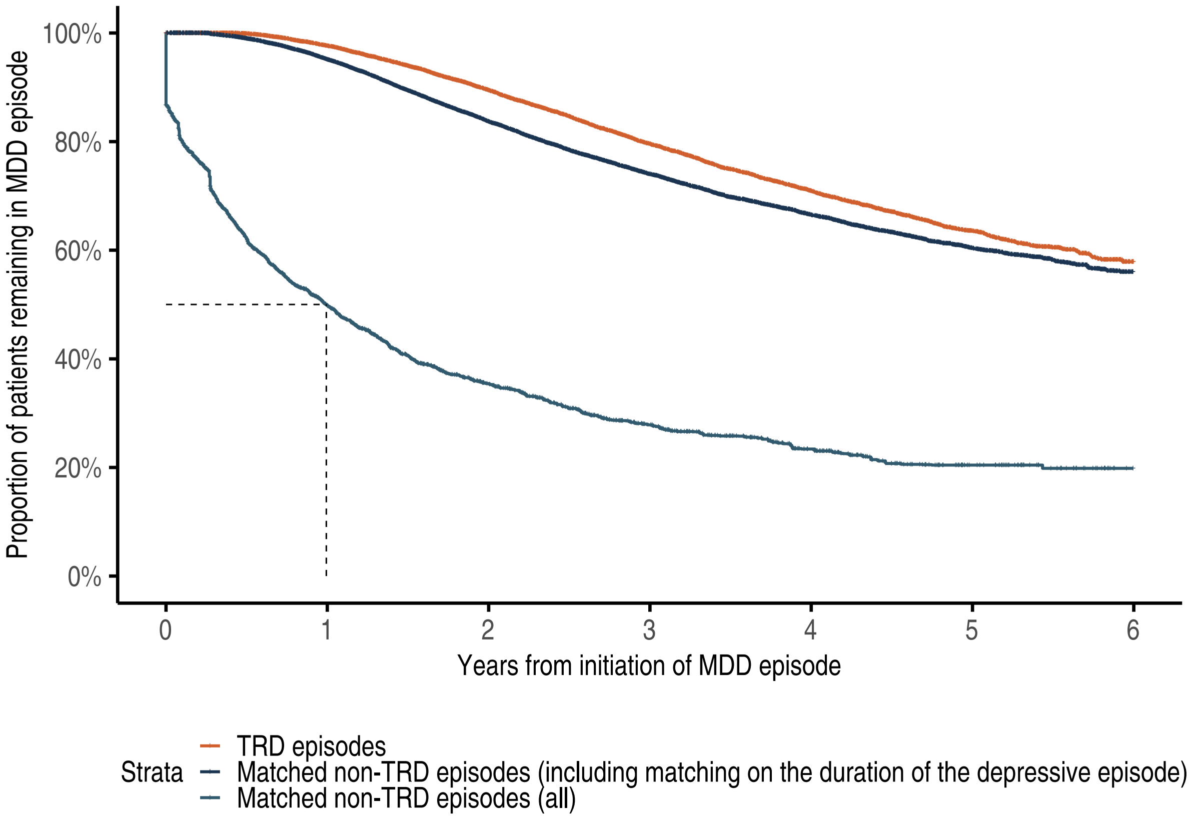

The figure presents the duration (or rather, the duration of healthcare contacts related to MDD) for the following three groups: (1) TRD episodes, (2) matched non-TRD episodes (where the matching procedure included the duration of the depressive episode in order to make this group more comparable with the TRD episodes), and (3) matched non-TRD episodes excluding the criterion of duration of the depressive episode. The motivation for this group (3) is to present the MDD duration for a more general MDD population.

All groups were followed from date of initiation of the MDD episode until the outcome (end of MDD episode), emigration from Stockholm, recordings of any of the exclusion diagnosis criteria (psychosis, dementia, manic episode, or bipolar disorder), death, or end of follow-up (December 31, 2018), which ever came first.

© 2022 Lundberg J et al. *JAMA Psychiatry.*

eFigure 7. Psychiatric comorbid conditions among TRD and matched non-TRD episodes

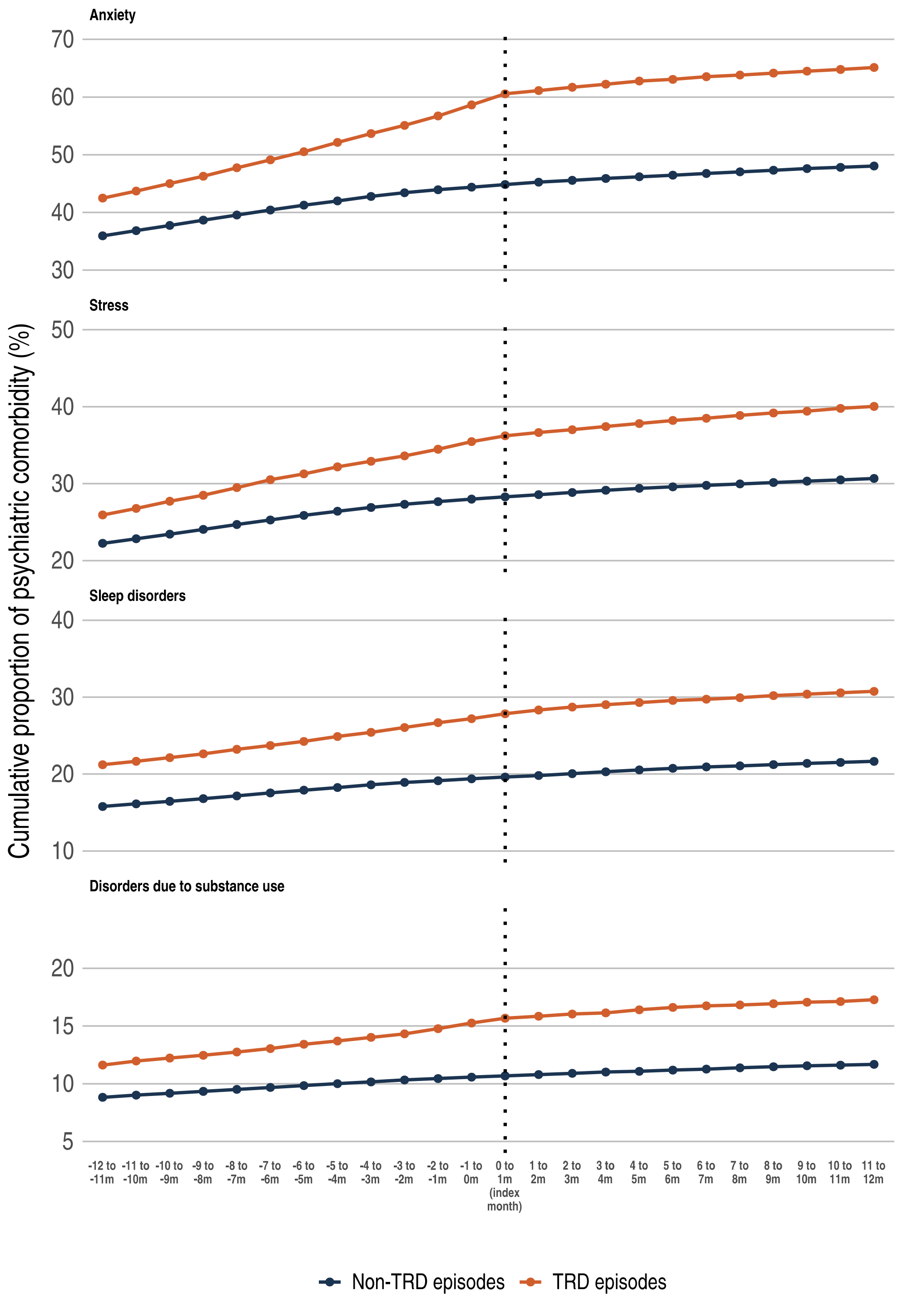

Figure 1 presents psychiatric comorbid conditions 12 months before and 12 months after index. Index for TRD episodes is the date when the MDD episode fulfills TRD criteria and non-TRD episodes were given the same index date as the matched TRD episode. In this cumulative analysis, patients were included the first month they were diagnosed with the psychiatric comorbid condition and carried forward in the analysis.

© 2022 Lundberg J et al. *JAMA Psychiatry.*

eFigure 8. Monthly healthcare resource utilization and lost work-days for TRD episodes compared to matched non-TRD episodes

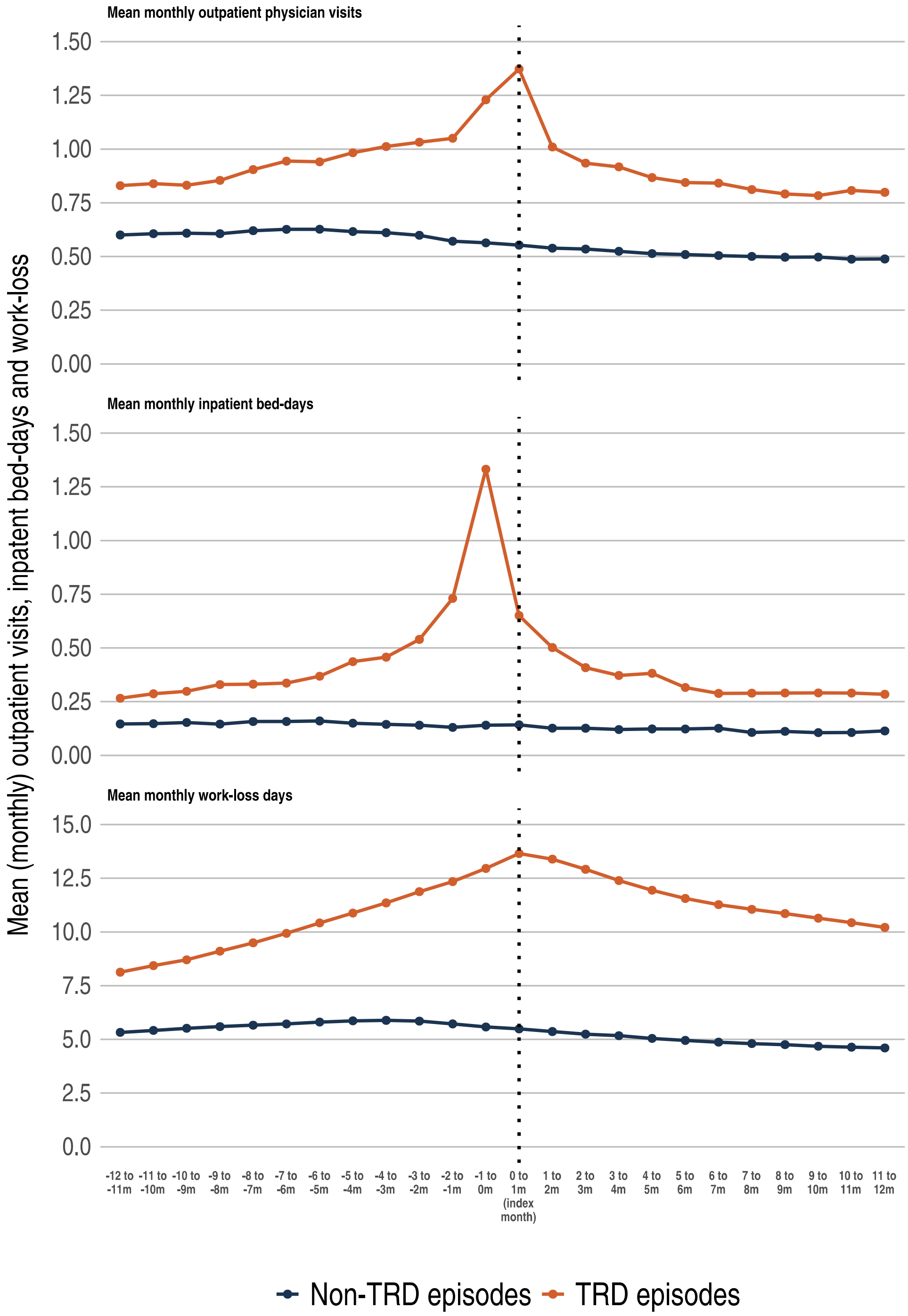

Figure 2 presents mean monthly healthcare resource utilization and work-loss 12 months before and 12 months after index. Index for TRD episodes is the date when the MDD episode fulfills TRD criteria and non-TRD episodes were given the same index date as the matched TRD episode.

eFigure 9. Treatments (on a substance level) when patients fulfill the TRD criteria (N=12 793)

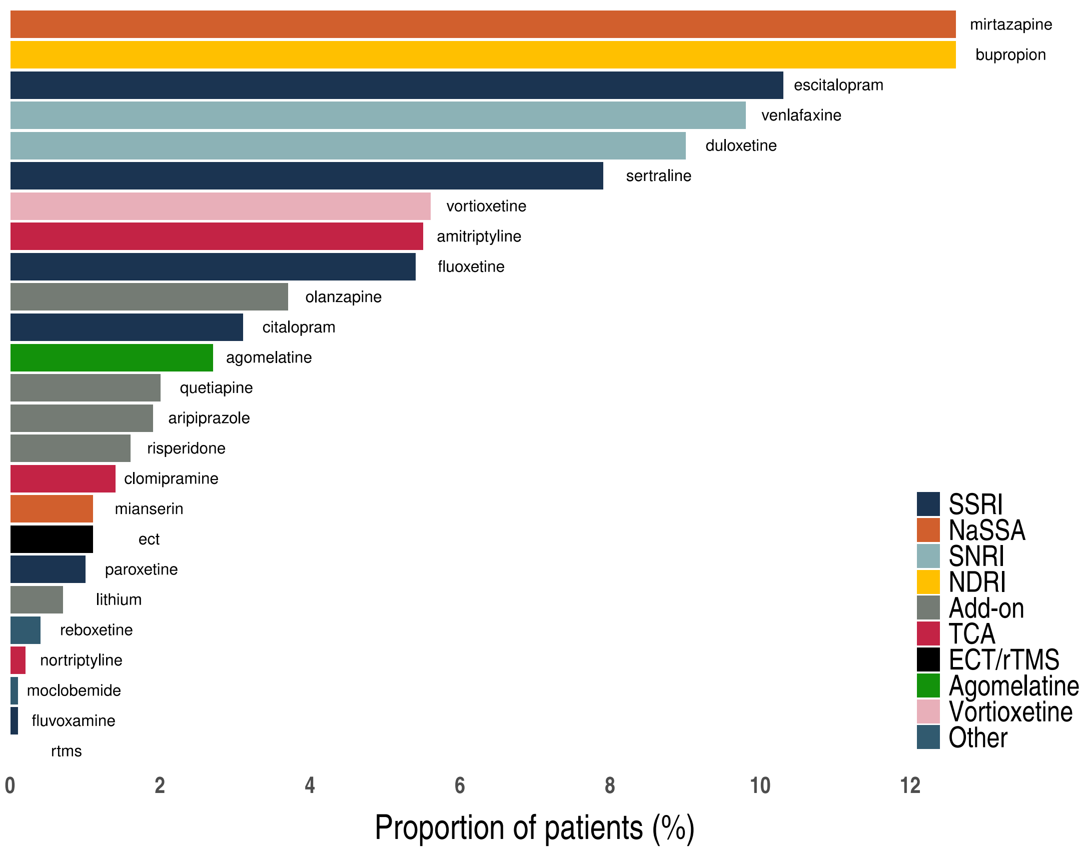

This bar chart presents the antidepressant therapy at time of fulfilment of TRD criteria (i.e. initiation of third antidepressant treatment trial within the depressive episode). The bars are colored according to the following therapeutic groups:

SSRI - selective serotonin reuptake inhibitors
NaSSA - noradrenergic and specific serotonergic antidepressant
SNRI - serotonin and norepinephrine reuptake inhibitors
NDRI - norepinephrine–dopamine reuptake inhibitor
ADD-ON – Add-on medication
TCA - tricyclic antidepressants
ECT/rTMS - electroconvulsive therapy or repetitive transcranial magnetic stimulation
Agomelatine
Vortioxetine
Other

For ATC codes (and clinical procedure codes) included in each group, see eTable 2.

© 2022 Lundberg J et al. *JAMA Psychiatry.*

eFigure 10. Calibration of the prediction model

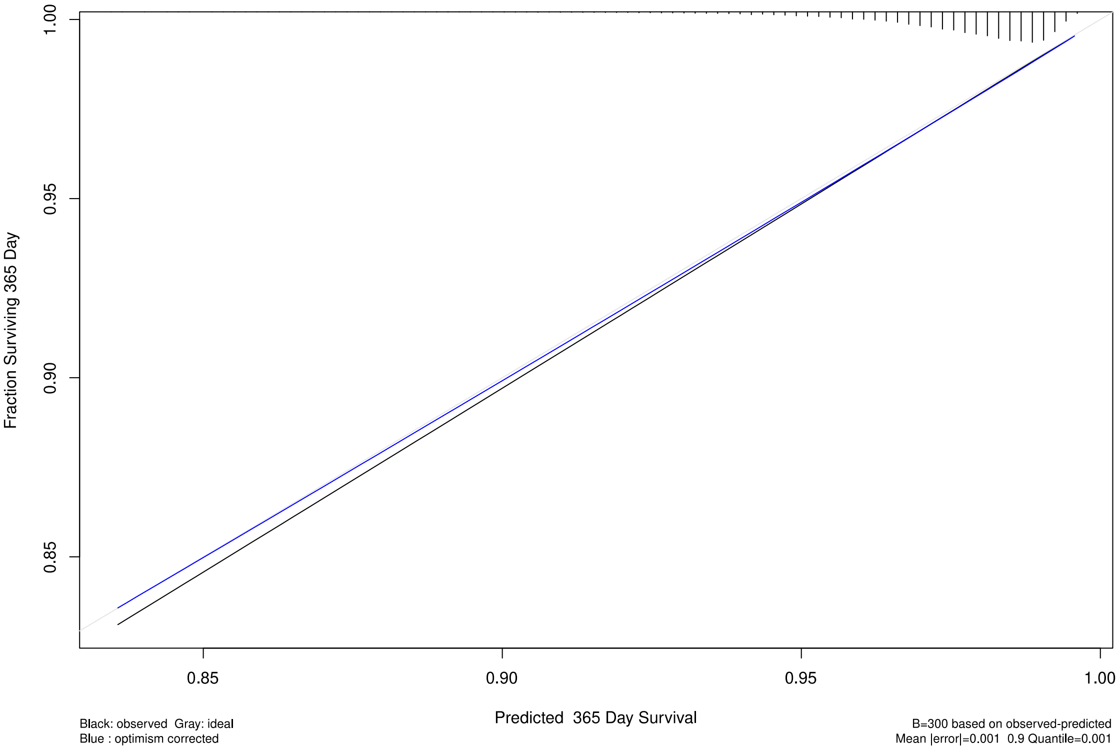

© 2022 Lundberg J et al. *JAMA Psychiatry.*

eFigure 11. Importance of each variable in the full model measured by partial χ^2 minus the predictor degrees of freedom

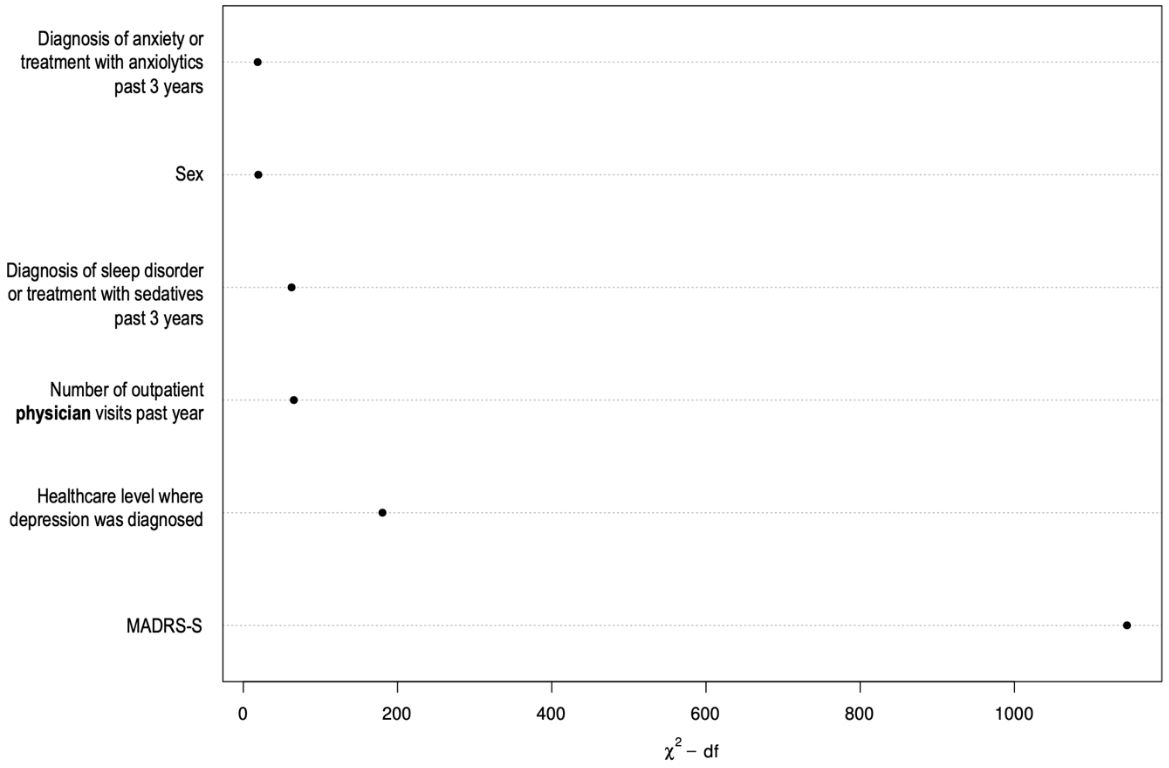

A higher value on the x-axis indicates greater importance for the prediction of TRD.

© 2022 Lundberg J et al. *JAMA Psychiatry.*
